# Novel regulation of miR‐34a‐5p and HOTAIR by the combination of berberine and gefitinib  leading to inhibition of EMT in human lung cancer

**DOI:** 10.1111/jcmm.15214

**Published:** 2020-04-05

**Authors:** Fang Zheng, Jing Li, ChangJu Ma, XiaoJuan Tang, Qing Tang, JingJing Wu, XiaoSu Chai, Jianhui Xie, Xiao‐bo Yang, Swei Sunny Hann

**Affiliations:** ^1^ Laboratory of Tumor Biology The Second Clinical College of Guangzhou University of Chinese Medicine Guangzhou China; ^2^ Department of Human Resource The Second Clinical College of Guangzhou University of Chinese Medicine Guangzhou China; ^3^ Department of Medical Oncology The Second Clinical College of Guangzhou University of Chinese Medicine Guangzhou China; ^4^ Guangdong Provincial Key Laboratory of Clinical Research on Traditional Chinese Medicine Syndrome The Second Clinical College of Guangzhou University of Chinese Medicine Guangzhou China

**Keywords:** E‐cadherin, EMT, HOTAIR, miR‐34a‐5p, NSCLC, Snail

## Abstract

HOTAIR is an important carcinogenic lncRNA and involves in tumorigenesis, and invasion. MiR‐34a‐5p functions as a tumour suppressor. However, the underlying mechanism of HOTAIR regulation especially in association with miR‐34a‐5p in non‐small‐cell lung cancer (NSCLC) has not been explored. Herein, we performed series of in vitro experiments, including viability, migration, invasion, apoptosis and in vivo xenograft model, and identified that HOTAIR was remarkably elevated in NSCLC cells. Enforced HOTAIR expression promoted migration and invasion, while depleted HOTAIR diminished the ability of migration and invasion of NSCLC cells. We also observed that miR‐34a‐5p was dramatically inhibited in NSCLC cells and the binding correlation between HOTAIR and miR‐34a‐5p was confirmed by dual‐luciferase reporter and RNA immunoprecipitation assays. We also showed that induction of miR‐34a‐5p and reduction of HOTAIR, and the interaction between miR‐34a‐5p and HOTAIR resulted in the suppression of epithelial‐mesenchymal transition (EMT) as illustrated by induction of key epithelial markers E‐cadherin expression, reduction of vimentin and EMT‐inducing transcription factor snail. Excessive expression of snail resisted miR‐34a‐5p‐inhibited cell growth. Snail binds to E‐cadherin promoter and regulates E‐cadherin expression. There was a synergy in combination of berberine and gefinitib in this process. Similar findings were also observed in a tumour xenograft model. Collectively, this is the first report demonstrating reciprocal interaction of miR‐34a‐5p‐ and HOTAIR‐mediated regulation of snail resulting in inhibition of EMT process by the combination of berberine and gefitinib suggesting that regulation of miR‐34a‐5p‐ and HOTAIR‐mediated inhibition of EMT may provide novel treatment paradigms for lung cancer.

## 1 INTRODUCTION

Lung cancer is one of the main causes of cancer‐related death worldwide. Despite advances in diagnosis and continuous development of novel and combination treatment modalities over the past decades, drug resistance is still one of the major obstacles for treatment efficacy in advanced non‐small‐cell lung cancer (NSCLC) and the underlying mechanisms still remains elusive.^1^, ^2^ Epidermal growth factor receptor tyrosine kinase inhibitors (EGFR‐TKIs) are currently the first‐line management of NSCLC patients with activating mutations within the kinase domain of the EGFR gene. However, these EGFR‐TKIs have eventually shown treatment failure due to the innate or acquired resistance including T790M mutation of EGFR, MET amplification, and phosphatase and tensin homolog (PTEN) deletion, among others.^3^, ^4^ Thus, much effort in searching for novel diagnostic and therapeutic approaches to improve treatment outcomes is greatly desirable.

The epithelial‐mesenchymal transition (EMT) is physiological and pathological reversible dynamic process in which epithelial cells change to structural and functional characteristics of mesenchymal cells with invasive potential.^5^ EMT contributed to enhance invasiveness and metastasis, resistance to treatment and development of cells with stem cell‐like characteristics.^6^ The hallmark of EMT was the loss of epithelial surface markers, such as E‐cadherin, and the acquisition of mesenchymal phenotype, which was associated with invasive or metastatic capacities.^5^ E‐cadherin is a transmembrane glycoprotein, and its functional loss has been strongly associated with poor overall survival in cancers.^7^ The dysregulation of E‐cadherin expression leading to carcinogenesis was occurred at both epigenetic and genetic levels, and enhanced E‐cadherin expression was involved in inhibition of growth, invasion and induction of cell cycle arrest and differentiation.^8^, ^9^ In lung cancer, miR‐598, as a tumour suppressor, reported to negatively regulate EMT thereby suppressing the invasion and migration in NSCLC cells through increasing E‐cadherin and decreasing vimentin expressions.^10^

Berberine (BBR), a quaternary proto‐berberine isoquinoline alkaloid extracted from plants, has been showed to possess anti‐tumour properties through distinct mechanisms.^11^, ^12^, ^13^, ^14^ Berberine hydrochloride inhibited proliferation and promoted apoptosis of NSCLC cells by suppression of the matrix metalloproteinase 2 (MMP‐2), Janus kinase 2 (Jak2)/vascular endothelial growth factor (VEGF)/nuclear factor κB (NF‐κB) regulatory axis.^15^ We previously showed that BBR inhibited growth and invasion of NSCLC cells through reduction of 3‐phosphoinositide‐dependent protein kinase‐1 (PDPK1) and transcription factor SP1 expressions; this resulted in a reduction of DNA methyltransferase 1 (DNMT1) expression. Metformin, a classical anti‐diabetic drug, strengthened the effects of BBR both in vitro and in vivo in lung cancer.^16^ Regardless, the detailed molecular mechanisms underlying this effect still remained undetermined.

Long non‐coding RNAs (lncRNAs), which are transcribed RNA molecules with longer than 200 nucleotides and have no obvious protein‐coding capacity, have been reported to regulate diverse biological processes, such as tumorigenesis, cell proliferation and apoptosis. Among these, HOX antisense intergenic RNA (HOTAIR) plays a central role in the pathogenesis of various tumours.^17^ Ectopic expression of HOTAIR has been involved in drug resistance, increased cell proliferation and migration through multiple signalling mechanisms in cancer.^17^ Thus, the importance of HOTAIR in cancer biology has sparked interest by using HOTAIR as a biomarker and potential therapeutic target.^18^, ^19^, ^20^ HOTAIR also played an important role in the prognosis of patients with NSCLC because the increased expression of HOTAIR was highly correlated with metastasis, drug resistance and shorten overall survival of patients.^18^, ^21^ Nevertheless, the significance and detailed mechanism underlying the true role of HOTAIR in the occurrence, growth and progression of lung malignancy still remained to be elucidated.

MicroRNAs (miRNAs) are small endogenous non‐coding RNAs that have been reported to regulate key biological functions in various physiological and pathological processes including initiation and progression of human cancers.^22^ Among these miRNAs, tumour‐suppressive miRNAs, such as miR‐34, have been found to suppress the expression of several oncogenes, such as *KRAS* and *c‐MYC* and to prevent and reverse tumorigenesis in vivo model of NSCLC.^23^ MiR‐34 is involved in pathogenesis of cancer by regulating downstream target genes, which could be considered as a biomarker for evaluating the prognosis of patients with cancer.^24^ This finding emphasized the importance of miR‐34 implicating in the tumorgenesis and development of cancer. While the links of HOTAIR and miR‐34a‐5p to the EMT process have been reported,^25^, ^26^, ^27^ the functional roles of HOTAIR and miR‐34a‐5p, and their interactions with EMT signalling pathways in lung cancer, especially in mediating the synergistic effects of berberine and gefinitib remain largely unknown.

In this study, we explored the potential synergistic responses of berberine and gefinitib in controlling human lung cancer cell growth, invasion and metastasis. Our results showed that the regulation of miR‐34a‐5p‐ and HOTAIR‐mediated inhibition of EMT and subsequently growth and metastasis by the combination of berberine and gefitinib  in human lung cancer.

## 2 MATERIALS AND METHODS

### 2.1 Cell lines and reagents

A549 and H1975 cells were provided by the Chinese Academy of Sciences Cell Bank of Type Culture Collection and authenticated for the absence of mycoplasma, genotypes, drug response and morphology by using a commercial kit provided by Guangzhou Cellcook Biotech Co. Ltd. All the cells were cultured in RPMI‐1640 medium (Gibco), supplemented with 10% (v/v) foetal bovine serum (Gibco), 100 µg/mL streptomycin and 100 U/mL penicillin (Gibco) at 37°C, with 5% CO_2_. In addition, the medium of A549‐Luc cells (carrying luciferase reporter gene obtained from the Guangzhou Land Biological Technology Co.) was added Geneticin (G‐418） Sulfate (Life Technologies) at concentration of 200 μg/mL. Gefitinib was purchased from Selleck Chemical (Batch No S102504, Purity, 99.93%), and berberine (98.01% of purity) was obtained from Chengdu Must Bio‐technology Company. Both drugs were prepared in dimethyl sulfoxide (DMSO) to obtain a stock solution of 10 mmol/L and 50 mmol/L and stored at −20°C. MTT powder was purchased from Sigma Aldrich. Monoclonal antibodies specific for Snai1, vimentin and E‐cadherin were purchased from Cell Signaling Technology Inc. Mimics and inhibitors of miR‐34a‐5p were obtained from Ribo Biological Co., Ltd.

### 2.2 Cell viability assay

The cell viability after treated with gefitinib and berberine were assessed by the 3‐(4, 5‐dimethylthiazol‐2‐yl)‐2, 5‐diphenyltetrazolium bromide (MTT) dye reduction method as described previously.^16^ Synergistic, additive or antagonistic effects of berberine and gefitinib combination treatment were classified by determining a combination index (CI) based on the Chou‐Talalay method ^28^ using CompuSyn software (Biosoft). The combination index (CI) values were calculated for each dose and the corresponding effect level, designated as the fraction affected (Fa) meaning the inhibited fraction of cell proliferation after drug administration. The CI values provide a quantitative definition for an additive effect (CI = 1), synergism (CI < 1) and antagonism (CI > 1) in drug combinations.

### 2.3 EdU incorporation assay

Cell proliferation was determined by Cell‐Light^TM^ EdU DNA cell proliferation kit obtained from Ribo Biological Co., Ltd. A549 and H1975 cells were seeded in 96‐well plates, after treated with berberine and gefitinib, the cells were exposed to 50 µmol/L of 5‐ethynyl‐2’‐deoxyuridine (EdU) for 2 hours at 37°C. Then, the cells were fixed in 4% PA‐PBS for 30 mins, and permeabilization with 0.5% TritonX‐100 for 10 mins. After that, the cells were stained with 1 × Apollo reaction regent for 30 minutes and stained the DNA contents with Hoechst 33342 for another 30 minutes. Subsequently, pictures were obtained under 400 × magnifications under microscopy (Nikon, TI2‐E). At least three captured fields were randomly selected and the EdU‐positive cells were calculated. The percentage of EdU‐positive cells = (EdU‐positive cells/Hoechst stain cells) × 100.

### 2.4 Cell migration and invasion assay

Cell migration was evaluated using wound healing assay. The cells were grown to more than 80% confluence in six‐well plates, then wounded with a sterile 200 μL pipette tip, washed with starvation medium to remove detached cells from the plates and treated with indicated doses of berberine and gefitinib in starvation medium. After 24 hours, the wound gap was observed and cells were photographed using an Olympus microscope fitted with digital camera. Cell invasion was measured using 24‐well Transwell plate (Corning) with 8 μmol/L pore size polycarbonate membrane. Before the experiment, cells were incubated overnight with starvation medium, diluted the Matrigel (BD) and injected into the upper chamber of Transwell system. The lower chamber was added into 500 μL of cell culture medium with 20% FBS. Then, 1x10^5^ cells were seeded into upper chamber, with indicated doses of berberine and gefitinib in starvation medium. After incubation for 24 h, cells, invaded were fixed in with 4% paraformaldehyde, stained with 0.1% crystal violet and counted under a microscope (Nikon, TI2‐E). Three independent experiments were carried out.

### 2.5 Flow cytometric analysis

Cell apoptosis was detected using Annexin V‐FITC/PI Apoptosis Detection Kit (BD Biosciences) according to the manufacturer's protocol. Briefly, H1975 cells were treated with BBR or gefitinib alone and combination of BBR or gefitinib for 24 hours, washed with pre‐cooled phosphate‐buffered saline (PBS) and trypsinized without the use of EDTA. Subsequently, the cells were harvested, resuspended in 500 μL of the binding buffer and incubated with 5 μL Annexin V‐FITC reagent for 15 minutes and 10 μL propidium iodide (PI) for 5 minutes at room temperature (RT) in the dark. Cell apoptosis was measured by flow cytometry (FC500, Beckman Coulter), and data were analysed by Graphpad Prism 5.0 software.

### 2.6 Western blot analysis

Equal amounts of proteins collected from different kinds of cell lysates were loaded on 10% SDS polyacrylamide gels using a vertical electrophoresis system (Bio‐Rad, Hercules, CA, USA) and then transferred onto PVDF membranes. The PVDF membranes were incubated with antibodies against Snai1, vimentin and E‐cadherin (1:1000) overnight at 4°C and then washed and incubated with a secondary antibody. The membranes were washed again and transferred to freshly made ECL solution (Millipore), followed by observing signals using the ChemiDoc XRS + Imagine System (Bio‐Rad). ImageJ software (National Institutes of Health) was used to quantify and compare the intensity of single band between the control and proteins of interest.

### 2.7 Quantitative RT‐ PCR

The total RNA was extracted by Trizol reagent (Roche). The reverse transcription was performed as described previously.^16^ RT‐PCR Primer Sets were purchased from Ribo Biological Co., Ltd. The sequences of primers are listed in Table. Quantitative real‐time PCR was performed in a 20 μL mixture containing 2 μL of the cDNA preparation, using FastStart Universal SYBR Green Master (ROX) Kit (Roche), on an ABI Quant Studio 7 Flex PCR System (Applied Biosystems). The PCR conditions were as follows: 10 minutes at 95°C, followed by 40 cycles of 10 seconds at 95°C and 60 seconds at 60°C. U6 or GAPDH were used as endogenous controls.

### 2.8 Transient transfection assays

HOTAIR small interfering RNA and control siRNA were purchased from GenePharma (GenePharma). The target sequences for HOTAIR siRNAs were as follows: si‐HOTAIR1, 5′‐GCCUUCCUUAUAAGCUCGU‐3′, 5′‐ACGAGCUUAUAAGGAAGGC‐3′; si‐HOTAIR2, 5′‐CAAUAUAUCUGUUGGGCGU‐3′, 5′‐ACGCCCAACAGAUAUAUUG‐3′; si‐HOTAIR3, 5′‐GGAAGCUCUUGAAGGUUCA‐3′ and 5′‐UGAACCUUCAAGAGCUUCC‐3′. The pcDNA3.1 and HOTAIR expression vectors purchased from GeneCopoeia Inc, were transfected into the cells using the lipofectamine 3000 reagent according to the manufacturer's instructions for up to 24 hours, followed by treating with berberine for an additional 24 hours. Mimics and inhibitors of miR‐34a‐5p were obtained from Ribo Biological Co., Ltd. and also transfected using the lipofectamine 3000 reagent.

### 2.9 Dual‐luciferase assay

The wild and mutation types of HOTAIR and Snai1 luciferase reporter constructs were designed and synthesized by GeneCopoeia, Inc. Co‐transfected these vector into the cells with either miR‐34a‐5p mimics or negative control using Lipofectamine 3000 Transfection Reagent; the preparation of cell lysis and measurement of luciferase activities were determined using the Luc‐Pair™ Dual Luminescence Assay Kit (GeneCopoeia). Luciferase activity was normalized with RLuc activity within each sample. In the separate experiments, E‐cadherin promoter plasmid with Renilla luciferase reporters as an internal control was purchased from GeneCopoeia. Snai1 overexpression plasmid (pEZ‐MO2‐snail) and the control vector purchased from GeneCopoeia were co‐transfected into the cells with the Lipofectamine 3000. After 24 hours, the cells were treated with berberine. A luciferase activity assay was performed using the Luc‐Pair™ Dual Luminescence Assay Kit (GeneCopoeia) according to instructions from the manufacturer. Each experiment was repeated at least three times in triplicate.

### 2.10 RNA immunoprecipitation (RIP) assay

The RIP assay was performed using the Magna RIP™ RNA‐Binding Protein Immunoprecipitation Kit (Millipore), according to the manufacturer's instructions. Briefly, the cells were rinsed and scraped with cold PBS, then lysed in complete RIP lysis buffer containing protease and RNase inhibitors. The cell lysis was incubated with RIP immunoprecipitation buffer containing magnetic beads conjugated with human anti‐AGO2 antibody (Millipore), and negative control IgG (Millipore, Billerica, MA, USA). Purified RNA was obtained and then applied to quantitative PCR with reverse transcription analysis. The expression of AGO2 was measured by Western blot.

### 2.11 Animal experiments

Animal studies were performed according to the protocols approved by the Institutional Animal Care and Use Committee of Guangdong Provincial Hospital of Chinese Medicine (the Ethics Approval Number 2018067) and the National Institutes of Health guide for the care and use of Laboratory animals (NIH Publications No. 8023, revised 1978). Six‐week‐old female BALB/c‐nu mice were purchased from Beijing Vital River Laboratory Animal Technology Co., Ltd. A total of 1 × 10^6^ A549‐Luc cells (carrying luciferase reporter gene obtained from the Guangzhou Land Biological Technology Co.) were injected subcutaneously near the axillary fossa region, or through tail vein injection. After one to two weeks of injection, the mice were then randomly assigned into four groups (10 mice/group for subcutaneous and 6 mice/group for tail vein injection) with or without 10 mg/kg berberine (purity 98.0%, Chengdu Herbpurify Co., Ltd), or 25 mg/kg gefitinib (purity 99.70%, MedChemExpress), or both, via intraperitoneal injection for up to 6 weeks. Tumour volume was calculated using the formula for a spheroid: volume = (width^2^ × length) × 0.5. Body weights of mice were measured once a week. After 3‐6 weeks, tumour bearing mice were killed, and xerografted tumours were harvested and prepared for the detection of the expressions of related genes and HOTAIR, miR‐34a‐5p via Western blot and qRT‐PCR analysis, respectively (Table 1). Also, mice lung were isolated and washed with PBS and fixed with 4% paraformaldehyde and embedded in paraffin and processed for haematoxylin‐eosin (H&E) stains (200 x). For bioluminescence imaging (BLI) procedure, each set of mice was injected with 150 mg/kg D‐luciferin (Caliper Life Sciences, Hopkinton, MA, USA). Imaging and quantification of signals (photons/sec) were controlled by the acquisition and analysis software in the IVIS‐200 imaging system (Xenogen Corporation, Berkeley, CA).

**Table 1:** The primer sequences of gene amplification by qRT‐PCR

Symbol	Primer	Primer Sequence(5’‐3’)
HOTAIR	Forward primer	5’‐GGTAGAAAAAGCAACCACGAAGC‐3’
	Reverse primer	5’‐ACATAAACCTCTGTCTGTGAGTGCC‐3’
GAPDH	Forward primer	5’‐CTCCTCCTGTTCGACAGTCAGC‐3’
	Reverse primer	5’‐CCCAATACGACCAAATCCGTT‐3’
miR‐34a‐5p	Forward primer	5’‐AGCCGCTGGCAGTGTCTTA‐3’
	Reverse primer	5’‐CAGAGCAGGGTCCGAGGTA‐3’
U6	Forward primer	5’‐ATTGGAACGATACAGAGAAGATT‐3’
	Reverse primer	5’‐GGAACGCTTCACGAATTTG‐3’

Note

The primer sequences of relevant gene amplification by qRT‐PCR were summarized.

### 2.12 Statistical analysis

All data are expressed as mean ± SEM. Statistical analyses were carried out using the unpaired, 2‐tailed Student's *t* test, differences between groups were assessed by one‐way or two‐way ANOVA and significance of difference between particular treatment groups was analysed using Dunnett's multiple comparison tests by GraphPad Prism software version 5.0. And statistical significance was assumed at a value of *P* < .05, asterisks showed in the figures indicate significant differences of experimental groups in comparison with the corresponding control condition.

## 3 RESULTS

### 3.1 Combination of berberine (BBR) and gefitinib  enhanced the inhibition of human lung cancer cell growth, migration and invasion, and induced apoptosis

**Figure 1:**
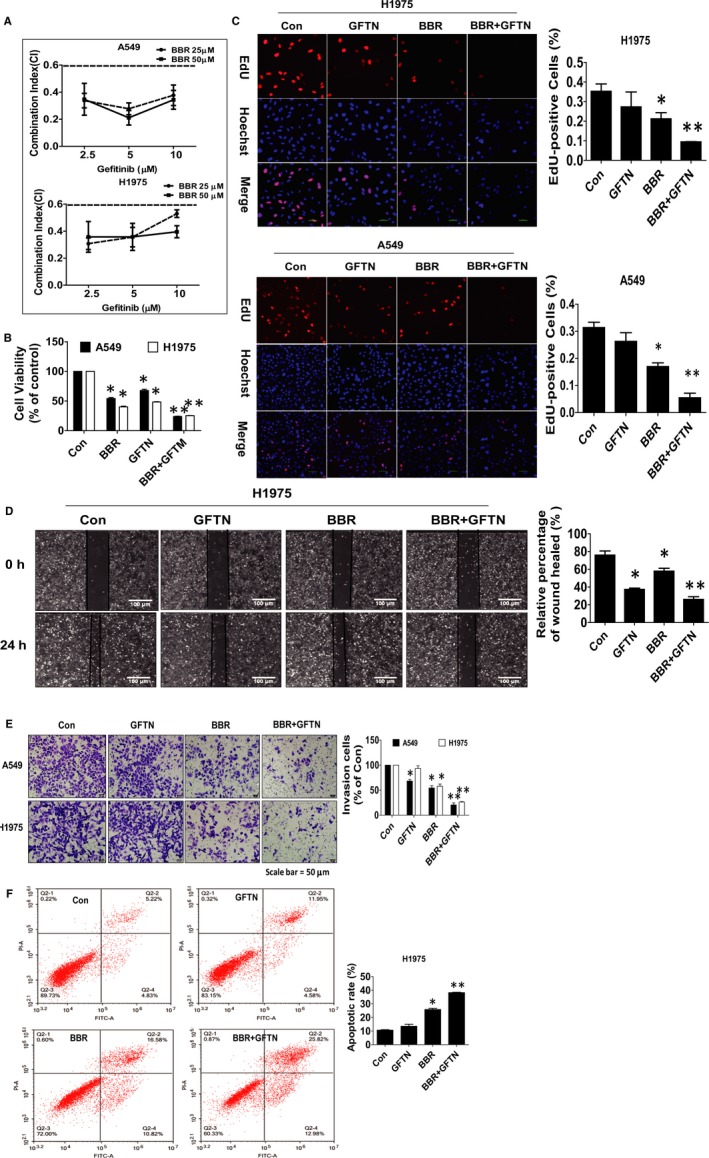
Combination of BBR and gefitinib  enhanced the inhibition of human lung cancer cell growth, migration and invasion, and induced apoptosis. A, B, A549 and H1975 cells were treated with indicated doses of BBR and GFTN for 48 h followed by measuring cell growth by MTT assays as described in the Materials and Methods section. The combination index (CI) values were calculated for each dose and the corresponding effect level, designated as the fraction affected (Fa) meaning the inhibited fraction of cell proliferation after drug administration. C, A549 and H1975 cells were treated with GFTN (5 μmol/L), BBR (25 μmol/L) and in combination one for 48 h followed by determination of cell growth with the Cell‐Light EdU DNA cell proliferation kit. The image was magnified 10×. Hoechst was used to stain all the nuclei. At least 5 captured fields were randomly selected, and the percentage of EdU‐positive cells = (EdU‐positive cells/Hoechst stain cells) × 100. Scale bars, 10 μm. D, E, A549 and H1975 cells were treated with GFTN (5 μmol/L), BBR (25 μmol/L) and in combination one for 48 h followed by measuring cell migration and invasion by wound healing and transwell assays as described in the Materials and Methods sections. Scale bars, 100 and 50 μm, respectively. F, H1975 cells were treated with GFTN (5 μmol/L), BBR (25 μmol/L) alone or combination of GFTN (5 μmol/L) and BBR (25 μmol/L) for 24 h, and then, cells were harvested for Flow cytometric analysis by using the Annexin V‐FITC/PI apoptosis kit. The E1 quadrant showed for percentage of dead cells, E3 quadrant represented percentage of normal cells, E2 and E4 quadrant indicated the percentage of late and early apoptosis, respectively. Values are given as the mean ± SEM from three independent experiments performed in triplicate. *indicates significant difference as compared to the untreated control group (*P* < .05). **indicates significant difference from the GFTN or BBR alone (*P* < .05)

Previous reports showed that BBR significantly inhibited the growth of NSCLC cells.^16^ In the current study, we further assessed the relative contribution to inhibition attributed to BBR and gefitinib in combination using Cell Proliferation BrdU (colorimetric) Kit (Figure 1A). We demonstrated that the combination of BBR with gefitinib  (EGFR‐TKIs) synergistically enhanced the inhibition of the proliferation of A549 and H1975 cells compared to treatment with BBR (25 μmol/L) or gefitinib  (5 μmol/L) alone (Figure 1A‐C). The CI values in cell growth inhibition were 0.28 ± 0.07 and 0.36 ± 0.07 in A549 and H1975 cells, respectively, suggesting moderate to strong synergy. Next, the function of BBR or gefitinib  in migration and invasion of NSCLC cells were determined via wound healing assays and transwell experiment, respectively. A549 and H1975 cells were treated with BBR, gefitinib  or in combination for 24 hours and then performed the wound healing and invasion experiments as described in the Materials and Methods section. The results showed that while BBR or gefinitib restrained the cell migration and invasive abilities of A549 and H1975 cells, there was an enhanced effect in the combination of BBR and gefitinib (Figure 1D‐E). In addition, we investigated the apoptosis affected by BBR, gefinitib or in combination using Annexin V‐FITC/PI Apoptosis Detection Kit. We showed that the combination of BBR and gefitinib  synergistically led to enhanced apoptosis at early stage compared with treated BBR or gefitinib  alone in H1975 cells (Figure 1F). Together, these results indicated the potential synergy of the BBR and gefinitib combination on the growth, migration, invasion inhibition and enhanced cell death in NSCLC including the EGFR‐TKI–resistant lung cancer cells.

### 3.2 BBR increased E‐cadherin, decreased vimentin and snail protein expressions, which were enhanced by the combination treatment in human lung cancer cells

**Figure 2:**
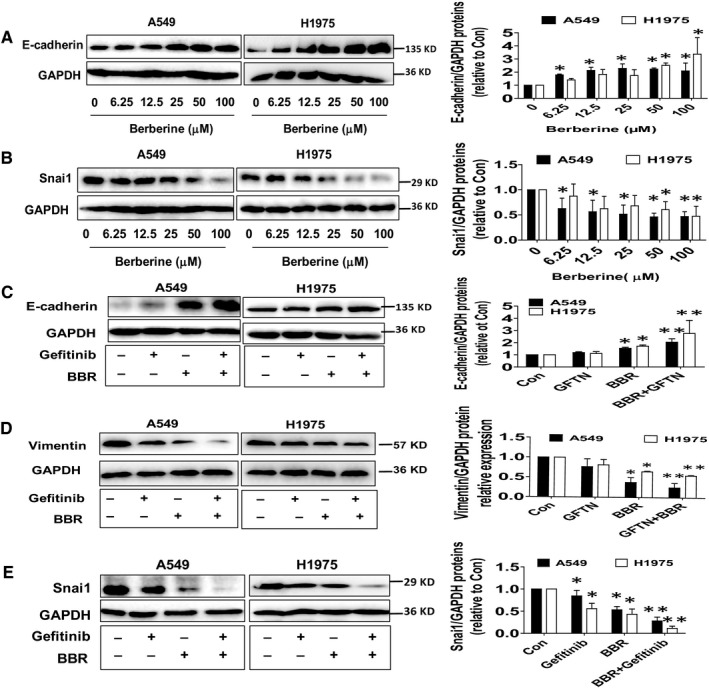
BBR increased E‐cadherin, decreased vimentin and snail protein expressions, which were enhanced by the combination treatment in human lung cancer cells. A, B, A549 and H1975 cells were treated with increased doses of BBR for 24 h followed by measuring E‐cadherin and snail protein levels via Western Blot. C‐E, A549 and H1975 cells were treated with GFTN (5 μmol/L), BBR (25 μmol/L) or in combination for 24 h followed by measuring E‐cadherin, vimentin and snail protein levels via Western Blot. GAPDH was used as loading control. The figures are representative cropped gels/blots that have been run under the same experimental conditions. Values are given as the mean ± SEM from three independent experiments performed in triplicate. *indicates significant difference as compared to the untreated control group (*P* < .05). **indicates significant difference from the GFTN or BBR alone (*P* < .05)

To explore the molecular mechanism underlying the synergistic enhancement in lung cancer cells, we began to investigate the potential molecules that may involve in this process. EMT is integral in development and wound healing, and contributes to cancer progression. This switch in cell differentiation and behaviour is marked by EMT‐associated markers including E‐cadherin, vimentin and mediated by key transcription factors, including snail.^29^ In this study, we observed that BBR increased E‐cadherin and decreased vimentin and snail protein expressions, which were enhanced by the combination treatment in human lung cancer cells (Figure 2A‐E).

### 3.3 BBR inhibited HOTAIR and increased miR‐34a‐5p expressions

**Figure 3:**
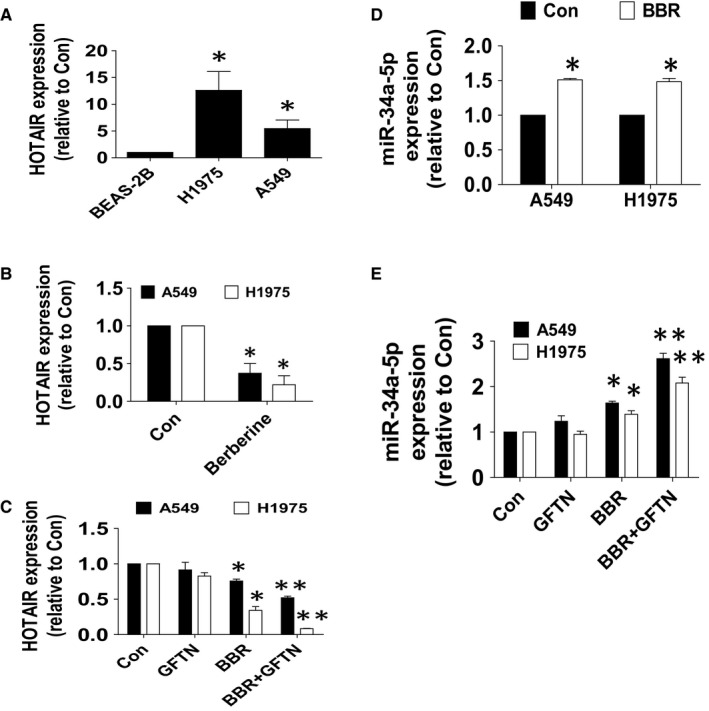
BBR inhibited HOTAIR and increased miR‐34a‐5p expressions. A, Total RNA was isolated from A549 and H1975 cells and normal human bronchial epithelial cells (BEAS‐2B) and processed for determining the mRNA levels of HOTAIR via qRT‐PCR. B, C, A549 and H1975 cells were treated with GFTN (5 μmol/L), BBR (25 μmol/L) or in combination for up to 24 h followed by measuring HOTAIR expression via qRT‐PCR. D, E, A549 and H1975 cells were treated with GFTN (5 μmol/L), BBR (25 μmol/L) or in combination for up to 24 h followed by measuring miR‐34a‐5p expression via qRT‐PCR. Values are given as the mean ± SEM from three independent experiments performed in triplicate. *indicates significant difference as compared to the untreated control group (*P* < .05). **indicates significant difference from the GFTN or BBR alone (*P* < .05)

To further dissect the mechanism underlying the EMT process, we started to explore the role of lncRNAs and miRNAs such as HOTAIR and/or miR‐34a‐5p, which were reported to be involved in the EMT process in different cancer types ^27^, ^30^ In that, we examined the effect of BBR on the expressions of HOTAIR and miR‐34a‐5p and found that relative high expression levels of HOTAIR was observed in lung cancer cells (A549 and H1975) compared with that in the lung epithelial cells (BEAS‐2B) (Figure 3A). BBR inhibited HOTAIR, and there was a synergy in the combination of BBR and gefitinib  (Figure 3B‐C). On the contrary, BBR induced expression of miR‐34a‐5p, which was enhanced in the combination of BBR and gefitinib  in lung cancer cells (Figure 3D‐E).

### 3.4 While exogenously expressed HOTAIR diminished BBR‐increased miR‐34a‐5p expression, the miR‐34a‐5p mimics inhibited HOTAIR expression and luciferase activity

**Figure 4:**
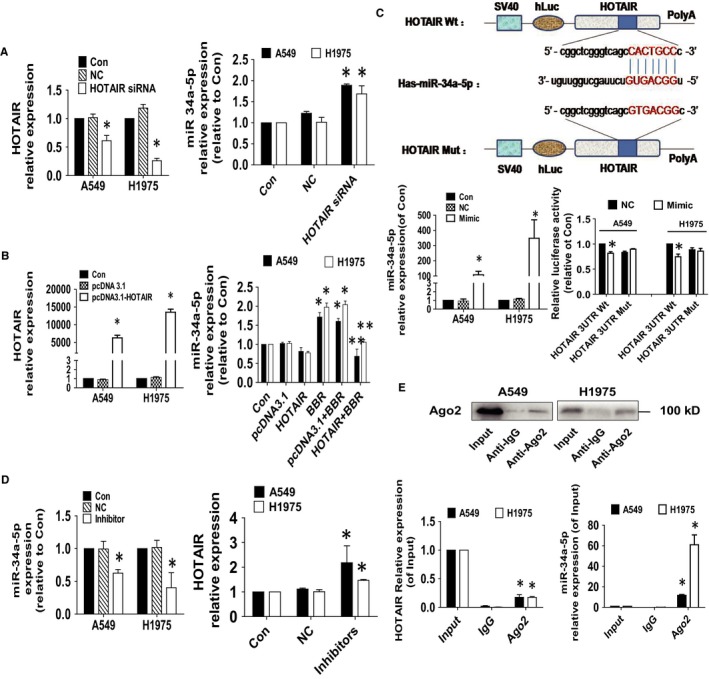
While exogenously expressed HOTAIR diminished BBR‐increased miR‐34a‐5p expression, the miR‐34a‐5p mimics inhibited HOTAIR expression and luciferase activity. A, B, A549 and H1975 cells were transfected with the control or HOTAIR siRNAs (25 nmol/L) or the control, HOTAIR expression vector for up to 48 h followed by measuring HOTAIR and miR‐34a‐5p expression via qRT‐PCR. C, The luciferase reporter constructs containing the wild‐type and mutant binding sites of HOTAIR were shown (upper panel). A549 and H1975 cells were transfected with the HOTAIR WT or HOTAIR Mut vectors (1.25 μg/mL each) for 24 h, then treated with the miR‐34a‐5p mimics (100 nmol/L) or miR‐negative control (NC) for an additional 48 h. Afterwards, the luciferase activity was detected using Secrete‐Pair™ Dual Luminescence Assay Kit as described in the Materials and Methods section (lower panel). D, A549 and H1975 cells were the miR‐34a‐5p inhibitors (100 nmol/L) or miR‐negative control (NC) for an additional 48 h followed by measuring HOTAIR and miR‐34a‐5p expression via qRT‐PCR. **E,** Cell lysates from A549 and H1975 cells were incubated with Ago2 antibody–coated magnetic beads. Precipitates ware subjected to Western blot for Ago2 protein and qRT‐PCR for detecting HOTAIR and miR‐34a‐5p expression levels. Preimmune IgG and input from cell extracts were used as controls. Values are given as the mean ± SEM from three independent experiments performed in triplicate. *indicates significant difference as compared to the untreated control group (*P* < .05). **indicates significant difference from the BBR alone (*P* < .05)

To further delineate the potential roles of HOTAIR and miR‐34a‐5p, we tested the potential interaction between HOTAIR and miR‐34a‐5p that mediated the effect of BBR on growth, migration and invasion. We observed that while silencing of HOTAIR enhanced, excessive expressed HOTAIR resisted the BBR‐induced expression of miR‐34a‐5p (Figure 4A‐B). Of note, overexpressed HOTAIR alone also showed slightly reduced expression of miR‐34a‐5p (Figure 4B). Furthermore, we found that the mimics of miR‐34a‐5p reduce the luciferase activity of HOTAIR in A549 and H1975 cells (Figure 4C). As expected, the inhibitors of miR‐34a‐5p stimulated the HOTAIR expression (Figure 4D). In addition, AGO_2_ RIP assays showed that HOTAIR could interact with miR‐34a‐5p (Figure 4E). Together, the above results indicated that BBR could regulate the expressions and reciprocal interaction of HOTAIR and miR‐34a‐5p in human lung cancer cells.

### 3.5 Overexpressed HOTAIR reversed BBR‐increased E‐cadherin protein expression; miR‐34a‐5p mimics not only reduced luciferase activity but also protein expression of snail

**Figure 5:**
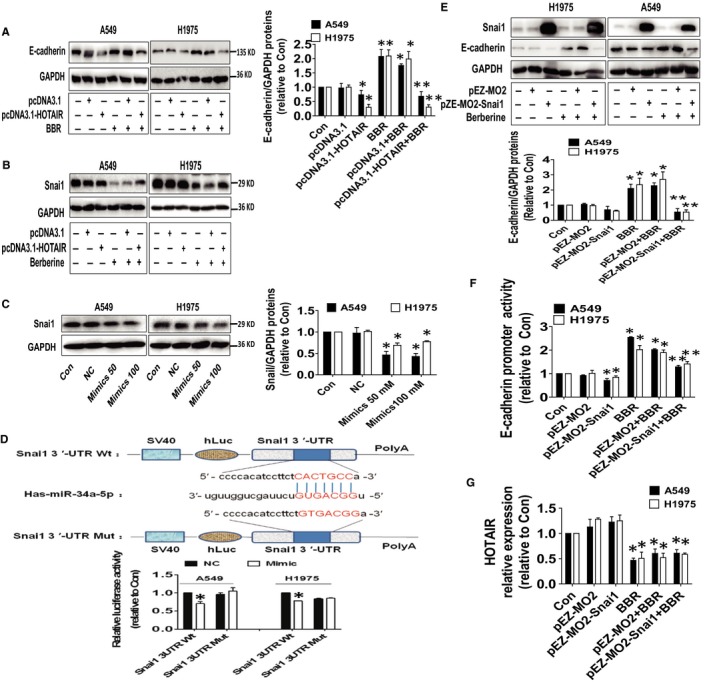
Overexpressed HOTAIR reversed BBR‐increased E‐cadherin protein expression; miR‐34a‐5p mimics not only reduced luciferase activity but also protein expression of snail. **A, B,** A549 and H1975 cells were transfected with the control and HOTAIR expression vector or 24 h before exposure the cells to BBR for an additional 24 h followed by measuring E‐cadherin and snail protein expressions via Western Blot. GAPDH was used as control. **C,** A549 and H1975 cells were transfected with the control and mimics of miR‐34a‐5p for 24 h followed by measuring snail protein expression via Western Blot. GAPDH was used as loading control. **D,** The luciferase reporter constructs containing the wild‐type and mutant binding sites in 3′‐UTR region of snail mRNA were shown (upper panel). A549 and H1975 cells were transfected with the snail 3’UTR‐WT or snail 3’‐UTR‐Mut vectors (1.25 μg/mL each) for 24 h, then treated with the miR‐34a‐5p mimics (100 nmol/L) or miR‐negative control (NC) for an additional 48 h. Afterwards, the luciferase activity was detected using Secrete‐Pair™ Dual Luminescence Assay Kit as described in the Materials and Methods section (lower panel). **E‐F,** A549 and H1975 cells were transfected with the control and snail expression vector, and E‐cadherin promoter plasmid, Renilla luciferase reporter internal control for 24 h before exposing the cells to BBR for an additional 24 h followed by measuring snail and E‐cadherin protein expressions and luciferase activity via Western Blot and Secrete‐Pair™ Dual Luminescence Assay Kit, respectively. GAPDH was used as loading control. The figures are representative cropped gels/blots that have been run under the same experimental conditions. **G,** A549 and H1975 cells were transfected with the control and snail expression vector for 24 h before exposing the cells to BBR for an additional 24 h followed by measuring HOTAIR expression via qRT‐PCR. Values are given as the mean ± SEM from three independent experiments performed in triplicate. *indicates significant difference as compared to the untreated control group (*P* < .05). **indicates significant difference from the BBR alone (*P* < .05)

To illustrate the functions of HOTAIR and miR‐34a‐5p in the EMT process affected by BBR, we further interrogate the relationships of HOTAIR and miR‐34a‐5p with EMT. We showed that overexpressed HOTAIR reversed BBR‐increased E‐cadherin protein expression (Figure 5A); snail, which is a zinc finger transcriptional repressor and has been linked to cancer biology, was reported a direct target of the miR‐34 family.^27^ We also observed that overexpressed HOTAIR overcame BBR‐reduced snail protein expression (Figure 5B). Of note, overexpressed HOTAIR alone had no significant effect on snail protein expression (Figure 5B). To further confirm and determine whether miR‐34a‐5p was able to directly inhibit snail expression, the 3′ UTR of snail was cloned downstream of a luciferase reporter construct. We showed that the mimics of miR‐34a‐5p not only reduce protein levels but also the luciferase activity in 3‐UTR region of snail (Figure 5C‐D). MiR‐34a‐5p‐dependent inhibition of the snail 3′‐UTR reporter activity was abolished after mutation of the predicted miRNA targeting sequences. Interestingly, exogenously expression of snail not only reduced but also resisted BBR‐induced E‐cadherin protein expression and promoter activity (Figure 5E‐F). Of note, we showed that exogenously expression of snail had no effects on HOTAIR implying little or no feedback regulatory loop existed in this process (Figure 5G). These results suggested that the accumulation of endogenous snail protein is increased in line with the inhibition of E‐cadherin expression.

### 3.6 *Anti‐tumour effect of BBR and erlotinib *in vivo* xerograft model*

**Figure 6:**
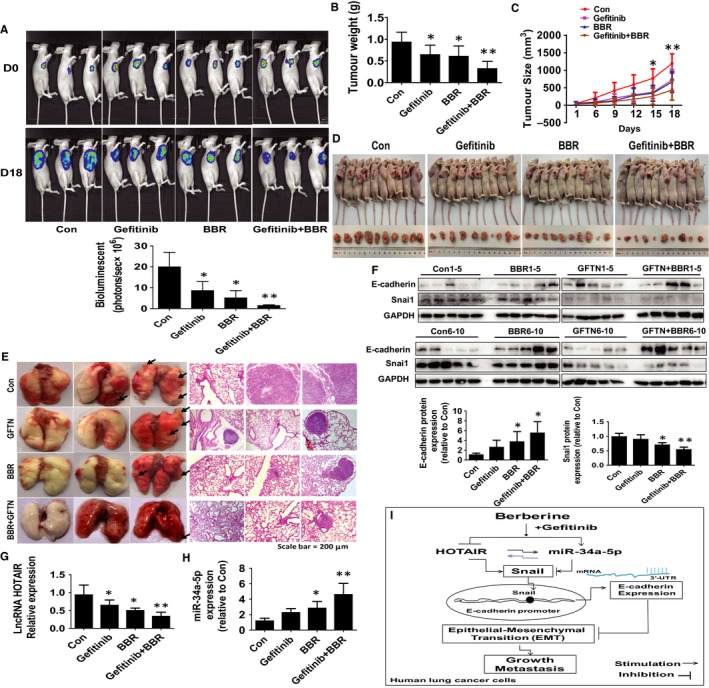
Anti‐tumour effects of BBR and gefitinib  in nude mouse xerograft model. A, The tumour growth was monitored by injecting luciferin followed by measuring bioluminescence signals. Representative images are shown. B, C, The xenografts were harvested on day 18, and the weight (B) and volume (C) of tumours were measured. D, The photographs of the vehicle‐treated, BBR, gefitinib and the concomitant treatment with gefitinib and BBR xenografts derived from nude mice were shown. E, Tail intravenously injected the tumour cells with aforementioned treatment into nude mice for 40 days and the gross lung images and H&E stains (100 x) of tumours were shown. F‐H, At the end of the experiments, the xenograft tumours were isolated and processed for detecting E‐cadherin and snail protein levels and expressions of HOTAIR and miR‐34a‐5p via Western blot and qRT‐PCR, respectively. The figures are the representative cropped gels/blots that have been run under the same experimental conditions. Values in bar graphs were given as the mean ± SEM from three independent experiments. *indicates significant difference as compared to the untreated control group (*P* < .05). **indicates significant difference from the BBR alone (*P* < .05). **I,** the diagram show that the reciprocal interaction of miR‐34a‐5p‐ and HOTAIR‐mediated regulation of EMT‐associated gene expression contribute to synergy of the combination of BBR and gefitinib  in human lung cancer cells. HOTAIR regulates the expression of snail through adsorbing miR‐34a‐5p, the latter targets and suppresses snail expression. By binding to E‐cadherin promoter region snail transcriptionally regulates E‐cadherin expression

In order to further verify our findings in vitro, we also tested the effects of BBR alone or in combination with gefitinib on lung tumour growth, the expressions of relevant proteins and miR‐34a‐5p, HOTAIR levels in the nude mouse xerograft model. We found that the mice treated with BBR showed a significant inhibitory effect on tumour growth as determined by the Xenogen IVIS200 system (Figure 6A). In addition, we observed a significant reduction of the tumour weight and sizes in the BBR and gefitinib treatment groups compared with the control group (Figure 6B‐D). More importantly, we observed potential synergy of BBR in combination with gefitinib (Figure 6B‐D). Moreover, we also tail intravenously injected tumour cells with aforementioned treatment into nude mice for 40 days before killing. The gross lung and H&E stains results showed a statistically significant decrease in tumour number, and size was observed in the treatment groups compared with the control group (Figure 6E), and the H&E stains of the lungs also indicated the BBR and combination‐treated lung found smaller and fewer tumours whereas the vehicle‐treated lung showed larger and more tumours (Figure 6E). Of note, in terms of bodyweight or physical conditions of animal used, we have not found any noticeable adverse effects from exposing BBR, gefitinib alone or in combination treatment groups during this study. In addition, consistent with the results in vitro*,* we found that increased E‐cadherin, decreased snail protein levels, inhibited HOTAIR and increased miR‐34a‐5p expressions were observed in the BBR and the combination groups as compared to that in the control one (Figure 6F‐H).

## 4 DISCUSSION

The EMT is the first step in the development of the invasive and migratory, and metastatic properties of cancer contributing to malignant tumour progression. The hallmark of EMT is the loss of epithelial surface markers, most notably E‐cadherin, and the acquisition of mesenchymal markers including vimentin and N‐cadherin.^5^ The suppression of E‐cadherin during EMT process can be mediated by transcriptional inhibition through binding of EMT transcription factors (EMT‐TFs), such as Snail, Slug and TWIST, to E‐cadherin promoter regions and regulated relevant functions, such as stability, localization, protein interactions, and ubiquitination.^31^, ^32^ The functional roles of HOTAIR and miR‐34a‐5p, and mechanistic analysis of their interplays with EMT signalling pathways in lung cancer, especially in mediating the synergistic effects of BBR and gefitinib  remain largely unknown. In this study, we explored the potential synergistic responses of BBR and gefitinib  in inhibition of human lung cancer cell growth. Our results showed that the regulation of miR‐34a‐5p‐ and HOTAIR‐mediated inhibition of EMT and subsequently growth and metastasis by the combination of BBR and EGFR‐TKI gefitinib  in lung cancer cells.

Long non‐coding RNA HOTAIR has been considered as a pro‐oncogenic factor in variety cancers.^33^ There is a growing body of evidence suggests that aberrantly expressed HOTAIR plays a critical role in the tumorgenesis, development and progression of multiple cancers.^33^ However, the precise mechanism remains largely elusive. MiRNA‐34 families (miR‐34s) are one of the well studied small non‐coding RNAs that regulate gene expression mainly at post‐transcriptional level. We investigated the function and mechanism of HOTAIR and miR‐34a‐5p in NSCLC cells and a xenograft mouse model. Our results indicated that opposite effect of HOTAIR and miR‐34a‐5p on NSCLC cell proliferation and invasion and involvement in mediating the enhanced anti‐lung cancer effect of BBR and gefinitib. The expression of HOTAIR was increased in NSCLC cells and involved in growth, invasion, metastasis and progression.^18^, ^34^, ^35^, ^36^, ^37^ The dysregulation of HOTAIR was correlated with metastasis and poor prognosis, and was expected to be considered as a potential biomarker and therapeutic target for patients with NSCLC.^38^ Thus, HOTAIR could be a potential target in cancer therapy. MiR‐34 was involved in the regulation of cell cycle and apoptosis through multiple signalling pathways. The anti‐oncogenic regulation of miR‐34s highlights the role in regulation of the growth, invasion, chemoresistance and sternness of cancer ^39^, ^40^ and was involved in pathogenesis in cancer by targeting different tumour‐related genes and signalling pathways.^39^, ^41^, ^42^ Our results suggested the tumour suppressor role of miR‐34a‐5p and further supported the use of miR‐34 as a potential novel preventative and anti‐tumour agent in lung cancer.

Our results also demonstrated and suggested that the interaction between HOTAIR and miR‐34a‐5p was involved in EMT process to repress tumorigenesis, cancer growth and metastasis, which have been reported in other studies.^30^, ^43^, ^44^, ^45^ We demonstrated that reductions of HOTAIR and inductions of miR‐34a‐5p have been involved in the BBR‐inhibited EMT process via inhibition of Snail. The transcription factor snail is a master regulator of cellular identity and a strong inhibitor of transcription of the E‐cadherin. Exogenously expressed snail caused tumorigenic and invasive properties in epithelial cells. Consistent with our findings, HOTAIR positively regulated snail expression by sponging miR‐148a thereby enhancing cell invasion and metastasis and promote the EMT in oesophageal cancer was reported.^46^ HOTAIR also mediated a physical interaction between snail and enhancer of zeste homolog 2 (EZH2), an enzymatic subunit of the polycomb‐repressive complex 2.^47^ The interaction among snail, HOTAIR and EZH2 on epithelial genes was found to be essential in the execution of the EMT highlighting the critical role of HOTAIR in regulation of snail in EMT suggesting that functional HOTAIR was required during snail‐induced EMT.^47^

As a zinc finger transcriptional repressor and a key regulator of EMT, the expression and regulation of snail have been reported to be involved in EMT process in cancer cells. Our results indicated a transcriptional regulation of E‐carherin by snail. In consistent with this, metformin, an AMP‐activated protein kinase (AMPK) activator, diminished the extracellular signal‐regulated kinase (ERK) signalling by activation of AMPK pathway, and this led to suppression of snail resulting in up‐regulation of a tumour suppressor and critical EMT marker E‐cadherin by binding to the E‐cadherin promoter. Metformin promoted EMT through an inverse interaction of AMPK and ERK signalling regulatory axis via regulation of E‐cadherin expression, which provide a potential for reducing cancer occurrence in metformin‐treated population.^48^

Moreover, our results unveiled a synergistic effect of combination of BBR and gefitinib  in the regulation of HOTAIR, miR‐34a‐5p and EMT, and lung cancer cell growth. These implied the potential new molecular mechanism underlying the combination of BBR and gefitinib  in controlling NSCLC cell growth. Substantial efficacy of BBR and other agents in combining has been reported to enhance potential anti‐tumour activities in other studies as well.^16^, ^49^ As the EGFR mutations and acquired resistance after long‐term treatment, combination therapy has become a promising strategy to overcome this drawback. Several efforts have been tried to evaluate the potential synergy of the combination therapy both in vitro and in vivo in NSCLC. The combination of EGFR‐TKI with other agents induced a more potent inhibitory effect against cancer.^50^, ^51^ BBR in combination of gefitinib showed a significantly enhanced effect on inhibiting proliferation, migration and invasion of lung cancer. More importantly, combinative treatment significantly inhibited the EMT and hence impeded the in vivo tumour development via down‐regulation of an oncogenic histone methyltransferase and gene transcriptional regulator EZH2 in lung cancer cells.^50^ Of note, the data obtained from BBR and gefitinib  combination treatment suggested potential synergy existed in our system; the clinical outcome of this combination therapy still needs to be evaluated. Nevertheless, the findings above might provide important information in combining use of natural anticancer compounds and other agents in increasing efficacy for the treatment of lung cancer.

Metastasis is the spread and growth of malignant cells at secondary sites inside the body**.** As one of the common in vivo metastasis assays, tail vein injection showed significantly lung metastatic lesions. Our in vivo data were consistent with the findings from that in vitro*,* confirming the strengthened effect of BBR in combination with gefitinib  on impeding lung tumour growth and regulation of snail and EMT marker expressions. The doses of BBR used were based on our and other reports.^16^, ^52^ Given the fact that the information of actual doses of BBR in animal blood remained underdetermined, more studies are needed to explore this. Additionally, whether BBR plays a role in increasing the survival in the experimental lung xenografted tumours required to be further explored.

Of note, we had not studied the potential upstream of HOTAIR, which may be direct target of BBR. The potential upstreams of HOTAIR, such as miR‐200c, thymic stromal lymphopoietin (TSLP) and c‐Myc, in regulation of HOTAIR thereby controlling invasion, tumorigenicity, proliferation and migration have been reported by others in different cell system.^53^, ^54^, ^55^ Also, direct target of BBR, such as nuclear receptor retinoid X receptor alpha (RXRα), in mediating the inhibition of cell growth was shown in colon cancer cells.^52^ We believe that exploring this using methods such as RNA immunoprecipitation (RIP) and/or RNA pull‐down, or 3' UTR luciferase reporter assays and identify the direct targets of BBR, which regulate HOTAIR expression, would further unveil the mechanism underlying the regulation and interaction of these molecules affected by BBR.

In conclusion, this is the first report demonstrating reciprocal interaction of miR‐34a‐5p‐ and HOTAIR‐mediated regulation of EMT‐associated gene expression by the combination of BBR and gefitinib  in human lung cancer cells. HOTAIR, as a competitive endogenous RNA (ceRNA), regulated the expression of snail through adsorbing miR‐34a‐5p, the latter targets and suppresses snail expression. By binding to E‐cadherin promoter region, snail transcriptionally regulates E‐cadherin expression. There was a synergy in combination of BBR and gefitinib  in this process (Figure 6I). Thus, this study suggests that therapeutic regulation of miR‐34a‐5p‐ and HOTAIR‐mediated inhibition of EMT may provide an opportunity to control NSCLC growth and metastasis by the combination of BBR and gefitinib .

## CONFLICT OF INTEREST

The authors declare that they have no conflict of interests.

## AUTHOR CONTRIBUTION

SSH conceived of the study, participated in its design and co‐ordination, draft and finalized the manuscript. FZ carried out the cell growth, siRNA, Western Blot assays, transfection and luciferase reporter assays. JL, CJM, XJT and QT participated in performed the cell viability, siRNA, transfection assays and protein expression experiments. JW and JX performed protein expression and statistical analysis. XY co‐ordinated and provided important suggestions including some reagents and critical reading the manuscript. All authors read and approved the final manuscript.

## Data Availability

The raw data supporting the conclusions of this manuscript will be made available by the authors to any qualified researcher.
